# Factors Associated with Enrolment of Households in Nepal’s National Health Insurance Program

**DOI:** 10.15171/ijhpm.2019.54

**Published:** 2019-07-07

**Authors:** Prabesh Ghimire, Vishnu Prasad Sapkota, Amod Kumar Poudyal

**Affiliations:** Central Department of Pubic Health, Institute of Medicine, Tribhuwan University, Kathmandu, Nepal.

**Keywords:** National Health Insurance Program, Health Insurance Board, Enrolment, Nepal

## Abstract

**Background:** Nepal has made remarkable efforts towards social health protection over the past several years. In 2016, the Government of Nepal introduced a National Health Insurance Program (NHIP) with an aim to ensure equitable and universal access to healthcare by all Nepalese citizens. Following the first year of operation, the scheme has covered 5 percent of its target population. There are wider concerns regarding the capacity of NHIP to achieve adequate population coverage and remain viable. In this context, this study aimed to identify the factors associated with enrolment of households in the NHIP.

**Methods:** A cross-sectional household survey using face to face interview was carried out in 2 Palikas (municipalities) of Ilam district. 570 households were studied by recruiting equal number of NHIP enrolled and non-enrolled households. We used Pearson’s chi-square test and binary logistic regression to identify the factors associated with household’s enrolment in NHIP. All statistical analyses were performed using IBM SPSS version 23 software.

**Results:** Enrolment of households in NHIP was found to be associated with ethnicity, socio-economic status, past experience of acute illness in family and presence of chronic illness. The households that belonged to higher socio-economic status were about 4 times more likely to enrol in the scheme. It was also observed that households from privileged ethnic groups such as Brahmin, Chhetri, Gurung, and Newar were 1.7 times more likely to enrol in NHIP compared to those from underprivileged ethnic groups such as janajatis (indigenous people) and dalits (the oppressed). The households with illness experience in 3 months preceding the survey were about 1.5 times more likely to enrol in NHIP compared to households that did not have such experience. Similarly, households in which at least one of the members was chronically ill were 1.8 times more likely to enrol compared to households with no chronic illness.

**Conclusion:** Belonging to the privileged ethnic group, having a higher socio-economic status, experiencing an acute illness and presence of chronically ill member in the family are the factors associated with enrolment of households in NHIP. This study revealed gaps in enrolment between rich-poor households and privileged-underprivileged ethnic groups. Extension of health insurance coverage to poor and marginalized households is therefore needed to increase equity and accelerate the pace towards achieving universal health coverage.

## Background

In many developing countries, out-of-pocket health expenditure of patients or their families constitute a large proportion of amount spent on healthcare. This proportion has been estimated to be the highest ie, 40.8% in the World Health Organization (WHO) South East Asia Region.^1^ In Nepal, household out-of-pocket health expenditure alone contributes to 56.3% of current health expenditure.^2^ In countries where out-of-pocket expenditure is the most important source of healthcare financing, households can experience financial catastrophe and often impoverishment as a result of their out-of-pocket spending on healthcare.^1,3^

Over the past decades, many low- and middle-income countries (LMICs) have faced severe challenges to sustain sufficient financing for healthcare and to provide adequate financial protection against impoverishing effects of catastrophic illness.^4^ Because of these concerns, moving away from out-of-pocket healthcare payments to prepayment social health protection mechanisms has widely been argued as an important step towards reducing risks from financial hardship. A resolution passed by world health assembly in 2005 called for countries to introduce prepayment mechanisms in the health sector for sharing risk as well as to avoid catastrophic healthcare expenditure and impoverishment of individuals as a result of seeking care.^5^ World health report 2010 also advocated health insurance as one of the promising means of subsidizing the entire population and achieving universal healthcare coverage.^6^ Various countries in the world responded to these calls by adopting different health financing mechanisms including voluntary community-based and social health insurance schemes.^7^

In Nepal, a variety of pre-paid healthcare financing schemes have been launched in the past to strengthen the social health protection of Nepalese citizens. Despite having a long history of private, non-profit health insurance schemes,^8,9^ a government funded community-based health insurance (CBHI) program was initiated in 2003 at 2 districts and was expanded to an additional 4 districts by 2006.^10^ Over the past several years, Ministry of Health made remarkable efforts to expand social health protection through health financing schemes such as Free Basic Healthcare Program, Aama program, Screening and Treatment of Uterine Prolapse and Poverty Stricken Citizens Fund.^10^ Nevertheless several of these schemes experimented in Nepal were often fragmented in resource allocation and inefficient in securing a comprehensive financial protection to its citizens.^11,12^ In this context, Government of Nepal recently embarked on a path to universal coverage through implementation of national social health insurance program. Consequently in 2016, a National Health Insurance Program (NHIP) was introduced in the country beginning its operation at 3 districts (Kailali, Ilam, and Baglung). By the mid of 2017, the program was operational in fifteen districts with gradual expansion to other districts in a phased manner.^13^

Nepal’s NHIP is a family-based scheme characterized by voluntary enrolment of households. In many LMICs, voluntary health insurance schemes have often failed to cover a large proportion of their target population.^14^ Even at national level, the government’s experience with implementing CBHI schemes in the past did not exhibit positive results. The sustainability of CBHI scheme was threatened by limited coverage of the population. The enrolment in public CBHI schemes ranged from 1.6%-12% of the catchment area population. The enrolment in private CBHI schemes was also lower ie, 2.7% of the population.^10^ The initial reports of NHIP also highlight difficulties in capturing its target population. Within the first year of its operation, only 5% of the population was covered by the scheme.^13^ Currently, there have been wider concerns regarding the capacity of NHIP to achieve adequate population coverage and remain viable. Nevertheless, Government of Nepal considers this scheme as a cornerstone for making progress towards universal coverage and aims to expand to all 77 districts by 2020. Given low enrolment in the scheme to date, achieving adequate coverage of households requires understanding factors that influence such enrolment.

Multitudes of factors are shown to have a variable influence on health insurance enrolment, and these factors vary between countries. Large body of literature from various LMICs suggest that enrolment in health insurance program is influenced by range of factors such as age, gender, and education of the household head, household income, household size, presence of children and elderly, place of residence, distance to health facility and household illness experience.^14,15^ However, only a limited number of studies in Asia have explored these factors at household level.^16,17^ Given a unique socio-economic context, health system status and a unique family-based insurance modality of the NHIP, the factors established at those countries are likely to vary in the Nepalese context. Nonetheless, the evidence base for health insurance programs in Nepal remains very weak. It is against this background that the study aimed to identify the factors associated with enrolment of households in the Nepal’s NHIP. The evidence generated might inform policy-makers towards addressing the problems of low enrolment in the NHIP.

### Overview of Nepal’s National Health Insurance Program

In order to ensure universal coverage, the government of Nepal adopted the National Health Insurance Policy in 2013. The policy aims to ensure equitable and universal access for all Nepalese citizens to necessary quality health services.^18^ Under this policy, a semi-autonomous Social Health Security Development Committee was established in 2015 (after the enactment of Health Insurance Act by the parliament of Nepal in 2017, Social Health Security Development Committee has been replaced by an autonomous Health Insurance Board) and the NHIP was rolled out at Kailali district in April 2016. The program was expanded to the 2 additional districts ie, Baglung and Ilam in June 2016^19^ and later the program was gradually expanded in a phase-wise manner to other districts. The government of Nepal aims to expand this program to all 77 districts by 2020.^13^ Premium for NHIP are collected from households and the annual contribution amount depends on the size of the family with Nepali Rupees (NPR) 2500 for families up to 5 members and NPR 425 for each additional member in the family. However, the payment of premium by ultra-poor, poor and marginalized groups are subsidized by the government.^20^ There is not any other cost sharing or co-payments with the NHIP, except the premium paid. Each family up to 5 members receives a benefit up to NPR 50 000. Families with more than 5 members receive an additional benefit of NPR 10 000 for each additional member of the family not exceeding a maximum benefit ceiling of NRP 100 000 per family.^13,20^ The benefit package of the NHIP consists of emergency services, outpatient consultations, inpatient services, selected drugs and diagnostic services. However, some services classified to be unnecessary or very expensive are on the exclusion list. Among these are cosmetic surgery, secondary equipment/machines such as artificial organs, vision glasses (costing more than NPR 500), hearing equipment, services relating to artificial insemination, abortion services, dental services and treatment for injuries resulting from fights or consumption of drugs or alcohol.^20^ NHIP is a cash-less system such that the members can receive services and drugs covered by the program without having to pay at any stage. The provider payments are made by Health Insurance Board on the basis of claims made by providers according to the agreed rates. The claim management process is streamlined through Insurance Management Information System (IMIS).^21^ Table 1 presents key features of the NHIP.

**Table 1:** Key Features of NHIP

**Features**	**Description**
Roll out year	- April 2016
Administration	- Currently administered by an autonomous Health Insurance Board (During the implementation of this study, the scheme was administered by Social Health Security Development Committee, a semi-autonomous body) The board provides membership cards, makes decisions on contribution amounts, develops mechanisms for subsidies to poor and disadvantaged groups, as well as negotiates with service providers on benefit package, their costs and deals with provider payments
Membership	- Voluntary scheme based on family contributions^20^
Sources of revenue	- Budget allocated by Government of Nepal - Premium contributions from households where families with up to 5 members contribute NPR 2500 (US$21.59^a^) per year and NPR 425 (US$3.67) per additional member^13,20^
Exemptions	- 100% exemption in annual premium for ultra-poor, 75% for poor and 50% for marginalized^20^
Service delivery channels	- Public Health Facilities - Private health facilities selected through contracting
Benefit Package	- Benefits of up to NPR 50 000 (US$431.70) per year are provided to insured families of up to 5 members with an additional NPR 10 000 (US$86.34) covered for each additional member. The maximum amount of benefit available to a family per year is NPR 100 000^20^ - Covers emergency services, outpatient consultations, selected inpatient services, drugs and diagnostic services - Includes 928 types of medicines^13^
Co-payments	- No co-payments or other cost sharing arrangements
Exclusion list	- Services considered to be unnecessary or very expensive are on the exclusion list - Excludes cosmetic surgery, spectacles costing more than NPR 500 (US$4.32), hearing aids, artificial insemination, dental services and treatment for injuries suffered in a drunken brawl^20^
Provider payment mechanisms	- Case-based payment for outpatient and emergency services - Fee for service for inpatient and diagnostic services
Claim management	- Service provider health institutions submit claim to Health Insurance Board through IMIS - Health Insurance Board reviews and approves the claim for reimbursement to service provider health institutions
Information system used	- Uses an internet-based IMIS. This system is used for registration of membership and renewal, claim management, feedback and reporting

Abbreviations: NPR, Nepali Rupees; NHIP, National Health Insurance Program; IMIS, insurance management information system. ^a^Note: 1 US dollar equivalent to 115.82 NPR based on exchange rate of Nepal Rastra Bank.^22^

## Methods

### Settings for the Study

This study was purposively conducted in Ilam district which was one of the first 3 districts (Kailali, Ilam, and Baglung) that had completed its first year of enrolment cycle. Ilam is a hill district of Eastern Nepal and 1 of the 14 districts of province 1. The district covers an area of 1707 km^2^ with population of 287 916.^23^ It is administratively divided into 6 Palikas (4 municipalities and 6 rural municipalities). Ilam Municipality and Sandakpur rural municipality were selected as study sites. Ilam municipality is district headquarter located at about 600 km east from Kathmandu, the capital city. Sandakpur rural municipality lies about 20 km north-east from district headquarter. Both Ilam and Sandakpur have a population of diverse ethnic groups comprising of privileged Brahmins, Chhetris, Gurungs and Newars and underprivileged janajatis (indigenous people) and dalits (the oppressed).^24^ For centuries, these underprivileged groups are socially ascribed lower in the caste/ethnicity hierarchy and face certain disparities in terms of access to healthcare, education, economic opportunities as well as political and social representation.^25^

### Study Design and Sampling Procedure

This study was cross-sectional and comparative. 570 households were studied by recruiting equal number of enrolled (n = 285) and non-enrolled (n = 285) households. The sample size was estimated using Epi Info StatCalc software assuming 90% power of the study and 95% level of confidence. We assumed the percentage of households from under-privileged group discontinuing the insurance scheme at 56% with an odds ratio of 1.76.^26^ The sample size for each Palika (municipality) was proportionate to the total number of NHIP enrolled households. The selection of enrolled households at each Palika was done using simple random sampling. The list of enrolled households was obtained from Social Health Security Development Committee (now Health Insurance Board) through its IMIS. Enrolment assistants (volunteers who are responsible to register and enrol families to NHIP) and female community health volunteers helped in locating the sampled households. For every enrolled household recruited for the study, one comparison household (not-enrolled to NHIP) was selected from the nearest neighbour located in any direction. Non-enrolled households living in the study area for less than 6 months were not included in the study.

### Data Collection

A structured questionnaire was developed based on study objectives. A Nepal Demographic and Health Survey questionnaire was adapted for measuring wealth index.^27^ In order to enhance the content validity of the tool, the questionnaire was subjectively assessed by 2 health insurance experts for its content, organization, appropriateness as well as logical flow of the instrument. Contextualization of the tool was done by reviewing national documents on health insurance. The questionnaire was pretested among 44 households before application. The questionnaire included 4 sections; socio-demographic and economic information; morbidity status, perceived health status of the family and enrolment status of households. Household survey to collect data was carried out from September to October 2017. The tool was administered to household heads in Nepali language using face to face interview. A written informed consent was obtained from the participants before interview. One enumerator with a university degree and prior field research experience was trained and mobilized as interviewer.

### Variables

### Outcome Variable

The outcome variable for this study was the health insurance enrolment status of households. Those enrolled in the NHIP were coded as 1 and 0 otherwise.

### Explanatory Variables

The choice of explanatory variables in this study was guided by Anderson and Newman behavioural model of health service utilization^28,29^ and the review of literature on the determinants of enrolment in the health insurance schemes.^30-35^ The behavioural model envisages that household’s decision to enrol in health insurance scheme depends on 3 set of factors namely; predisposing factors (demographic, and social structures and health beliefs that predisposes households to enrol in the NHIP), enabling factors (that facilitate or impede households to enrol in the NHIP) and need factors (that induce the need for households to enrol in the NHIP).

In this study, the predisposing factors included age of household head classified into 3 categories (less than 40 years, 40-59 years and 60 years or older); gender of household head (male or female); education of household head (no formal education, up to secondary level education or post-secondary education); household size (≤5 members or >5 members); family type (nuclear or joint/extended); presence of children aged 0-5 years (no children or at least one children); presence of elderly above 60 years (no elderly or at least one elderly); number of family members who had completed their secondary education (none or at least one member) and ethnicity (privileged or underprivileged). Privileged ethnic group comprised of upper caste people (Brahmin and Chhetri) and relatively advantaged janajatis (Newar and Gurung) and underprivileged ethnic group comprised of dalits and disadvantaged janajatis. The socio-economic status of households was examined as a predisposing factor. This was measured by constructing a wealth index using principal component analysis based on data on household ownership of durable assets. The components included were ownership of house, electronic assets (television, refrigerator, computer/laptop), mobile and non-mobile telephone, vehicles, animals, types of fuel used for cooking and source of drinking water. These components were converted into a weighted index (factor score) and the households were then divided into 5 quintiles of wealth.^36^ The first quintile represented the poorest segment of the population and fifth quintile, the least poor. Needs factors in this study included morbidity conditions of households and perceived health status of the family. Morbidity conditions of households was assessed using 2 variables; past illness experience and presence of chronic illness in the family. Past illness experience was a self-reported response in which respondents were asked to recall if any of their family members had experienced any acute illness requiring health facility visit within 3 months preceding the survey. The responses were recorded as “Yes” or “No.” The presence of chronic illness was recorded as “Yes” if at least one of members in family suffered from either of heart disease, diabetes, chronic obstructive pulmonary disease, and cancer. Perceived health status of family was rated as good, average, or poor. Table 2 presents a summary of the study variables.

**Table 2:** Summary of Study Variables

**Variable**	**Definition of Variables**	**Measurements**
**Outcome Variable**		
Enrolment status	Status of the household’s membership in the NHIP	Enrolled (coded 1), not-enrolled (coded 0)
**Explanatory Variables**		
Age	Age of household in completed years at the time of household survey	Less than 40 years, 40-59 years, 60 years or older
Gender	Gender of household head	Male, female
Education	The highest level of education attained by household head	No formal education, up to secondary level, post-secondary education
Ethnicity	Ethnicity of household members	Privileged (upper caste people and advantaged janajati), under-privileged (dalits and disadvantaged janajati)
Household size	The number of family members living in the household	Five or less members, more than 5 members
Family type	Type and composition of family	Nuclear, joint or extended
Presence of children aged 0-5 years	The number of children aged between 0 to 5 years present in the household	None, at least 1 child
Presence of elderly above 60 year	The number of elderly members above 60 years present in the household	None, at least 1 elderly
No. of members completed secondary education	The number of family members who have completed their secondary education	None, at least 1 member
Socio-economic status	Socio-economic position of households based on wealth quintile	Richest, rich, middle, poor, poorest
Illness experience	Number of family members who experienced any acute illness within 3 months prior to the survey period	None, at least 1 member
Presence of chronic illness	Number of family members who have at least one chronic illness	None, at least 1 member
Perceived health status of the family	Health status of the family as rated by the household head	Good, average, poor

Abbreviation: NHIP, National Health Insurance Program.

### Data Management and Analysis

A digital questionnaire was prepared using Epi Info application and data were collected using tablets. To prevent the risk of data loss, the collected data was uploaded to cloud storage on a daily basis. Compilation of data was done in Epi Info^TM^ 7.2.1.0 and then exported to IBM SPSS Statistics version 23.0 for cleaning and analysis. Descriptive statistics were used to report the demographic and socio-economic factors, morbidity characteristics and perception. The wealth index was generated using principal component analysis. Pearson’s chi-square test was performed to test the association of independent variable with enrolment in NHIP. Multiple logistic regression was used to investigate the effect of these variables on the odds of enrolment in the NHIP. Multi-collinearity of variables was tested before entering them in the regression analysis. No problem of collinearity was seen among the variables (the highest observed VIF was 1.937). All variables significant at 15% significance level in bivariate analyses were considered for multiple logistic regression.^38^ The goodness of fit of regression model was tested by the application of Hosmer and Lemeshow chi-square test; the model was found to be a good fit (*P* > .05).

The regression model was explained by the equation:

Log [Y/(1-Y)] = *b*_o_ + *b*_1_*X*_1_ + *b*_2_*X*_2_ + *b*_3_*X*_3_.....*b*_n_*X*_n_ + e

Where *Y* is the expected probability for the outcome variable to occur, *b*_o_ is the constant/intercept, *b*_1_ through*b*_n_ are the regression coefficients and the *X*_1_ through *X*_n_ are distinct independent variables and e is the error term.

## Results

### Characteristics of the Study Population

The mean age of respondents was 41.8 (standard deviation [SD] = 13.5 years). Majority (87.4%) of the households were headed by males. While more than one in 3 household heads (35.3%) had completed their secondary level education, nearly 1 in 5 household heads (18.6%) had no formal education. The average household size of the study population was 4.8 (SD = 1.6).

More than half of the surveyed households belonged to privileged ethnic group (56.1%). Majority households had equal to or less than 5 members (74.4%) and lived in a nuclear family (54.7%). Slightly less than one-third households (30.2%) had children below 5 years of age and about 2 in 5 households (39.6%) had elderly members above 60 years of age. Three in 4 households (76.3%) had at least 1 family member who had completed their secondary education. Two in 5 households (43.5%) had at least 1 family member with illness experience in past 3 months. More than one-third households (36.1%) had a family member suffering from chronic illness. Slightly more than one in ten household heads (13.5%) perceived health status of their family as poor (Table 3).

**Table 3:** General Characteristics of the Study Population (n = 570)

**Characteristics**	**Number**	**Percent**	**95% CI**
Age^a^			
Less than 40 years	275	48.2	44.1-52.4
40-59 years	224	39.3	35.3-43.4
60 years or older	71	12.5	9.9-15.5
Gender			
Male	498	87.4	84.4-90.0
Female	72	12.6	10.0-15.6
Education of household head			
No formal education	106	18.6	15.5-22.0
Up to secondary level (grade 1-10)	263	46.1	42.0-50.3
Post-secondary education (>grade 10)	201	35.3	31.3-39.3
Ethnicity			
Privileged ethnic group	320	56.1	52.0-60.3
Underprivileged ethnic group	250	43.9	39.7-48.0
Household size^b^			
Five or less members	424	74.4	70.6-77.9
More than 5 members	146	25.6	22.1-29.4
Family type			
Nuclear	312	54.7	50.5-58.9
Joint or extended	258	45.3	41.1-49.5
Presence of children aged 0-5 years			
None	398	69.8	65.9-73.6
At least 1 child	172	30.2	26.4-34.1
Presence of elderly above 60 years			
None	344	60.4	56.2-64.4
At least one elderly	226	39.6	35.6-43.8
Illness experience (in past 3 months)			
None	322	56.5	52.3-60.6
At least one member	248	43.5	39.4-47.7
Presence of chronic illness			
None	364	63.9	59.8-67.8
At least one member	206	36.1	32.2-40.2
Perceived health status of family			
Good	275	48.2	44.1-52.4
Average	218	38.2	34.2-42.4
Poor	77	13.5	10.8-16.6

^a^Mean ± standard deviation [SD] = 41.8 ± 13.5. ^b^Mean ± SD = 4.8 ± 1.6.

### Factors Associated With Enrolment in NHIP

In the bivariate analyses of enrolment in NHIP with demographic and socio-economic factors, morbidity characteristics and perception, significant association was found with education of household head (*P*  = .001), household size (*P*  = .035), type of family (*P*  < .001), ethnicity (*P*  < .001), presence of elderly (*P*  = .001), number of members with completed secondary education (*P*  < .001), socio-economic status (*P*  < .001), illness experience in family (*P*  < .001), and presence of chronic illness (*P*  < .001). Gender of household head, presence of children and perceived health status of family did not show any significant association with enrolment of households in NHIP (Table 4).

**Table 4:** Association of Enrolment in NHIP With Various Characteristics

**Demographic, Socio-economic, Morbidity Characteristics and Perception**	**Enrolled (n = 285)**		**Not-enrolled (n = 285)**		***P*** **Value**
	**No. (%)**	**95% CI**	**No. (%)**	**95% CI**	
Gender of household head					.313
Male	253 (50.8)	46.3-55.3	245 (49.2)	44.7-53.7	
Female	32 (44.4)	32.7-56.6	40 (55.6)	43.4-67.3	
Education of household head					.001^a^
Secondary level and higher	120 (59.7)	52.6-66.5	81 (40.3)	33.5-47.4	
Below secondary level	165 (44.7)	39.6-49.9	204 (55.3)	50.1-60.4	
Household size					.035^a^
More than 5 members	84 (57.5)	49.1-65.7	62 (42.5)	34.3-50.9	
Five or less members	201 (47.4)	42.6-52.3	223 (52.6)	47.7-57.4	
Family type					<.001^a^
Joint/Extended	150 (58.1)	51.9-64.2	108 (41.9)	35.8-48.1	
Nuclear	135 (43.3)	37.7-49.0	177 (56.7)	51.0-62.3	
Ethnicity					<.001^a^
Privileged ethnic group	191 (59.7)	54.1-65.1	129 (40.3)	34.9-45.9	
Underprivileged ethnic group	94 (37.6)	31.6-43.9	156 (62.4)	56.1-68.4	
Presence of children aged 0-5 years					.584
At least 1	83 (48.3)	40.6-56.0	89 (51.7)	44.0-59.4	
None	202 (50.8)	45.7-55.8	196 (49.2)	44.2-54.3	
Presence of elderly above 60 years					<.001^a^
At least 1	132 (58.4)	51.7-64.9	94 (41.6)	35.1-48.3	
None	153 (44.5)	39.1-49.9	191 (55.5)	50.1-60.9	
No. of members completed secondary education					<.001^a^
At least 1	236 (54.3)	49.4-59.0	199 (45.7)	41.0-50.6	
None	49 (36.3)	28.2-45.0	86 (63.7)	55.0-71.8	
Socio-economic status					<.001^a^
Q1-Poorest	39 (34.2)	25.6-43.7	75 (65.8)	56.3-74.4	
Q2-Poor	61 (41.5)	33.4-49.9	86 (58.5)	50.1-66.6	
Q3-Middle	32 (41.5)	31.8-55.3	42 (56.8)	44.7-68.2	
Q4-Rich	62 (52.5)	43.1-61.8	56 (47.5)	38.2-56.9	
Q5-Richest	91 (77.8)	69.2-84.9	26 (22.2)	15.1-30.8	
Illness experience (in past 3 months)					<.001^a^
At least 1 member	149 (60.1)	53.7-66.2	99 (39.9)	33.8-46.3	
None	136 (42.2)	36.8-47.8	186 (57.8)	52.2-63.2	
Number of members with chronic illness					<.001^a^
At least 1 member	134 (65.0)	58.1-71.5	72 (35.0)	28.5-41.9	
None	151 (41.5)	36.4-46.7	213 (58.5)	53.3-63.6	
Perceived health status of family					.214
Good	144 (52.4)	46.3-58.4	131 (47.6)	41.6-53.7	
Average	99 (45.4)	38.7-52.3	119 (54.6)	47.7-61.3	
Poor	42 (54.5)	42.8-65.9	35 (45.5)	34.1-57.2	

Abbreviation: NHIP, National Health Insurance Program. ^a^Statistically significant at *P*  < .05.

During the regression analysis, enrolment in NHIP showed significant association with ethnicity, socio-economic status, illness experience in family and presence of chronic illness. The odds that households would enrol in NHIP were higher among those with higher socio-economic status. Richest households were 4 times more likely to enrol in NHIP (adjusted odds ratio [AOR]: 4.08, 95% CI: 2.15-7.72) compared to those in a poorest category. Similarly, households belonging to the privileged ethnic group were 1.7 times more likely (AOR: 1.71, 95% CI: 1.18-2.48) to enrol in NHIP compared to ones from underprivileged ethnic group. The households in which at least one of their members experienced acute illness were 1.5 times more likely (AOR: 1.51, 95% CI: 1.04-2.19) to enrol in NHIP compared to households that did not have such illness experience. Similarly, households with chronically ill members were 1.8 times more likely (AOR: 1.84, 95% CI: 1.23-2.73) to enrol in NHIP compared to households that did not suffer a chronic illness (Table 5).

**Table 5:** Odds of Enrolment in NHIP Due to Various Characteristics

**Demographic, Socio-economic and Morbidity Characteristics**	**Enrolment in NHIP**	
	**OR**	**95% CI**
Education of household head		
Secondary level and higher	1.37	0.90-2.07
Below Secondary level	1	1
Household Size		
More than 5	0.97	0.58-1.62
Less than or equal to 5	1	1
Family type		
Joint/extended	1.41	0.86-2.31
Nuclear	-	1
Ethnicity		
Privileged group	1.71^a^	1.18-2.48
Underprivileged group	1	1
No. of elderly above 60 years		
At least 1	1.11	0.71-1.74
None	1	1
No. of members completed secondary education		
At least 1	1.18	0.73-1.92
None	1	1
Socio-economic status		
Richest	4.08^a^	2.15-7.72
Rich	1.53	0.86-2.75
Middle	1.27	0.67-2.40
Poor	1.08	0.63-1.86
Poorest	1	1
Illness experience (in last 3 months)		
At least 1 member ill	1.51^a^	1.04-2.19
None	1	1
Presence of chronic illness		
At least 1 member chronically ill	1.84^a^	1.23-2.73
None	-	1

Abbreviations: OR, odds ratio; NHIP, National Health Insurance Program. ^a^Statistically significant at *P*  < .05.

## Discussion

Our study results showed that belonging to privileged ethnic group, having a higher socio-economic status, having an experience of acute illness by family member and presence of chronic illness in the family are the potential factors that influence the enrolment of households in NHIP.

This study confirmed an association between ethnicity and household enrolment in the NHIP. The households from privileged ethnic groups were more likely to enrol in NHIP compared to those from underprivileged ethnic groups. This might be explained by the fact that underprivileged ethnic groups are more likely to be financially unstable and have relatively less access to information and services. Hill janajati and hill dalits represent significantly higher proportions of the poor in Nepal.^39^ Paying for enrolment into the social security schemes like NHIP might therefore be too difficult for these groups. Previous studies have also confirmed the role of ethnicity in determining the enrolment of households in the health insurance schemes.^32,40,41^ Evidences from studies in various LMICs have shown a positive association between wealth or socio-economic status and health insurance uptake.^34,35,42,43^ Bivariate and multivariate analyses in this study also confirmed a similar association. The richest households were 4 times more likely to enrol in NHIP as compared to those in poorest category. One explanation for these findings might be that richer and ethnically privileged families are better connected to the government and enrol more in other non-healthcare related government programs and services. With greater interactions, they might have better exposure to insurance information and knowledge on how to enrol. In a study about United States Medicaid program, Saavedra showed that those enrolled in other forms of government programs are more likely to have health insurance coverage.^44^

Despite the policy provisions to subsidize the premium of the poor and marginalized,^20^ NHIP does not seem to provide financial protection to these segments of population. The study results clearly show marked differences in enrolment status between rich-poor and privileged-underprivileged ethnic groups. These findings point out the issues of inequity in enrolment. This might be because the pro-poor targeting have not yet been realized in practice due to the delay in distribution of poverty identification cards to these segments of the population.^13^ Addressing these disparities in enrolment of household in the NHIP across socio-economic and ethnic group is necessary to accelerate the pace towards achieving universal health coverage.

This study found an association between experience of acute illness and enrolment in the NHIP. The presence of chronically ill member in the household also showed significant association with NHIP enrolment. Similar findings have also been reported in several studies from other LMICs.^17,45,46^ These findings support the notion that families with pre-existing health conditions or more prone to being ill have a greater tendency to enrol in a health insurance scheme. From a public health perspective, this is very encouraging as it enhances healthcare access for those with poor health.^17,47^ The observed association also indicates the possibility of adverse selection taking place in a NHIP which is critical from a sustainability point of view. Adverse selection results when high-risk or sick individuals enrol more in the health insurance schemes compared to low-risk or healthy individuals. Adverse selection might limit potential for cross-subsidies and can affect the sustainability and financial viability of the scheme.^17,48^

In the case of Nepal’s NHIP, entire households are enrolled as unit. Household enrolment is ideally believed to lessen the problems of adverse selection by bringing into the insurance pool all healthier family members those who would not otherwise enrol.^49^ Nevertheless, the financial viability of the scheme can be threatened if the provisions for household enrolment are not strictly enforced. In a study of rural mutual healthcare health insurance scheme in China, Wang et al found significant adverse selection among partially enrolled households because the policy to enrol households as a unit was not fully enforced.^50^ Similar situation in Nepal’s NHIP therefore cannot be ruled out where there are also possibilities for larger size households to partially enrol their sickest member. Since the partial enrolment status of households was not examined by our study, we recommend detailed studies to substantiate the presence of adverse selection and to assess whether it would threaten the financial viability of the scheme.

Considering the patriarchal nature of Nepalese society, men are conventionally considered responsible for major financial decisions within the households. In contrast to this presumption, gender of household head in this study showed no association with enrolment in NHIP. The available evidence however is mixed regarding association between gender of household head and enrolment in health insurance scheme. While some studies suggested that female headed households are more likely to purchase health insurance,^31,34,40^ others have shown that male headed households are more likely to enrol in health insurance program,^35,51^ and yet other studies reported no significant association in enrolment in health insurance schemes between male and female headed households.^32,52^

Although factor such as education of household head was significantly associated in bivariate analysis, this relationship was not significant after adjustments using regression analysis. However, the role of education of household head in enrolling their families into health insurance schemes cannot be ruled out. Studies from Bangladesh, Ghana, Burkina Faso, and Zimbabwe have shown positive associations between health insurance uptake and higher education of household head.^32,33,35,46,53^ Also our study did not find any significant association between poor perceived health status and increased uptake of health insurance although other studies have established such association.^34,54^

The findings of this study might be relevant to policy and decision-makers interested in increasing the NHIP enrolment rates for households. First, the policy-makers should consider the fact that poor socio-economic households are less enrolled to NHIP than households with higher socio-economic status. Simply proclaiming subsidies for poor and marginalized households may not be enough to induce these households to enrol into the NHIP. Robust and timely measures might be necessary in order to put the pro-poor targeting policy into practice. Second, policy-makers may wish to ensure that households from underprivileged ethnic groups have as much access to health insurance program as families from privileged ethnic groups. Health Insurance Board and policy-makers need to design policies and interventions that will ensure equitable enrolment of unreached marginalized ethnic groups. At the meantime, local governments and other community stakeholders could play a significant role to raise insurance awareness, engender community trust and increase the connectedness of households to government programs and schemes.

To the best of our knowledge, this study provides an early evidence on the factors associated with household enrolment in the newly implemented health insurance program of Nepal. Although the findings of this study are consistent with large body of literatures from various LMICs, we could not relate these findings to our national context owing to the limited number of literatures available for Nepal. The results of this study might be affected by the purposive selection of the study sites. Furthermore, the selection of neighbours of enrolled households for comparison might have introduced a selection bias. Due to the cross-sectional nature of our data, it was not possible to demonstrate a temporal relationship between enrolment in NHIP and explanatory variables. Despite these limitations, the results of the study will add to the knowledge base on the NHIP and generally be useful to policy- and decision-makers at the government and health insurance board and those in academia.

## Conclusion

Belonging to the privileged ethnic group, having a higher socio-economic status, experiencing an acute illness and presence of chronically ill member in the family are the factors associated with enrolment of households in NHIP. Our study revealed gaps in enrolment between rich-poor households and privileged-underprivileged ethnic groups. Despite the stated pro-poor targeting of the NHIP, there is a clear gap between the policy and its practice. Extending health insurance coverage to poor and marginalized households is therefore needed to increase equity and accelerate the pace towards achieving universal health coverage.

## Acknowledgements

This study was funded by the University Grants Commission under it Masters Research Support Program to the first author (Award no. MRS/74_75/HS-6). Department of Community Medicine and Public Health, Maharajgunj Medical Campus provided an institutional support to complete this study. The authors also acknowledge the support of Health Insurance Board. We thank Keshab Sanjel and Narayan Budhathoki for their editorial assistance. We are grateful to the study participants, field enumerator who contributed to data collection and enrolment assistants and female community health volunteers who helped in locating the sampled households.

## Ethical issues

The research protocol was approved by Institutional Review Board at Institute of Medicine, Tribhuwan University, Kathmandu, Nepal.

## Competing interests

Authors declare that they have no competing interests.

## Authors’ contributions

PG designed the study, acquired data, performed data analysis and drafted the manuscript. Both VPS and AKP participated in the conception and design of the study and provided important critical revisions of the manuscript for important intellectual content. AKP provided critical inputs during statistical analysis. All authors read and approved the final manuscript.

## Key messages

**Implications for policy makers**

A National Health Insurance Program (NHIP) was designed to ensure equitable access to healthcare by all Nepalese citizens. This paper raises questions about how to ensure equitable participation of poor and ethnically disadvantaged families into the risk pool of NHIP.

- In this study, households with poor socio-economic status enrolled less as compared to richer families. Health insurance board and government must create robust measures to timely identify and subsidize poorer households as stipulated in the national health insurance policy.
- Results also show that enrolment in NHIP is disproportionally concentrated among the privileged ethnic groups. Hence, Health Insurance Board and policy-makers need to design policies and interventions that will ensure equitable enrolment of marginalized ethnic groups.
- The results of this study indicated the presence of adverse selection in the scheme. More detailed studies are recommended to substantiate the occurrence of adverse selection in NHIP and assess the effect it would have upon its financial viability.

**Implications for public**

Pre-existing poor health condition of family member and/or being prone to illness have found to influence household’s enrolment in National Health Insurance Program (NHIP). This study suggests a need for all households regardless of their illness status to enrol in the scheme in order to ensure better protection against uncertain financial consequences resulting from any impending catastrophic illnesses. Local governments and other community stakeholders also need to play significant roles to raise insurance awareness, engender community trust and increase connectedness of households to government programs and schemes such that all segments of population in their communities are equitably enrolled to the scheme irrespective of ethnicity, economic or morbidity status.

## References

[1] WHO. World health statistics 2016: monitoring health for the SDGs sustainable development goals: Geneva: World Health Organization; 2016.

[2] Pandey J, Karna R, Shrestha D, Neupane G, Bajracharya B. Nepal national health accounts, 2009/2010–2011/2012. Kathmandu, Nepal: Ministry of Health, Government of Nepal; 2016.

[3] Saito E, Gilmour S, Rahman MM, Gautam GS, Shrestha PK, Shibuya K (2014). Catastrophic household expenditure on health in Nepal: a cross-sectional survey. Bull World Health Organ.

[4] Gottret P, Schieber G. Health financing revisited: a practitioner’s guide. The World Bank; 2006.

[5] WHO. Sustainable health financing, universal coverage and social health insurance, Resolution WHA58.33. Geneva: World Health Organization; 2005.

[6] WHO. The world health report 2010: health systems financing: the path to universal coverage. Geneva: World Health Organization; 2010. 10.2471/BLT.10.078741 PMC2878164 20539847

[7] Giedion U, Andrés Alfonso E, Díaz Y. The impact of universal coverage schemes in the developing world: a review of the existing evidence. 2013.

[8] Ghimire R. Community based health insurance practices in Nepal. International Research and Reviews. 2013;2(4).

[9] An inventory of micro-insurance schemes in Nepal. Kathmandu: International Labour Office in Nepal, International Labour Organization; 2003.

[10] Stoermer M, Fuerst F, Rijal K, et al. Review of community-based health insurance initiatives in Nepal. Deutsche Gesellschaft fur internationale Zusammenarbeit (GIZ) Gmbh; 2012.

[11] Pokharel R, Silwal PR (2018). Social health insurance in Nepal: A health system departure toward the universal health coverage. Int J Health Plann Manage.

[12] Torres LV, Gautam GS, Fuerst F. Assessment of the government health financing system in Nepal: suggestions for reform. Deutsche Gesellschaft für Internationale Zusammenarbeit; 2011.

[13] Annual Report for FY 2073/74. Kathmandu: Social Health Security Development Committee; 2017.

[14] Acharya A, Vellakkal S, Taylor F, et al. Impact of national health insurance for the poor and the informal sector in low- and middle-income countries. London: The EPPI-Centre;2012.

[15] Dror DM, Hossain SS, Majumdar A, Koehlmoos TLP, John D, Panda PK (2016). What factors affect voluntary uptake of community-based health insurance schemes in low- and middle-income countries? a systematic review and meta-analysis. PLoS One.

[16] Kamath R, Sanah N, Machado LM, Sekaran VC (2014). Determinants of enrolment and experiences of Rashtriya Swasthya Bima Yojana (RSBY) beneficiaries in Udupi district, India. Int J Med Public Health.

[17] Alkenbrack S, Jacobs B, Lindelow M (2013). Achieving universal health coverage through voluntary insurance: what can we learn from the experience of Lao PDR?. BMC Health Serv Res.

[18] Government of Nepal. National health insurance policy. Kathmandu: Ministry of Health and Population; 2013.

[19] Ministry of Health. Annual Report for 2015/2016. Kathmandu: Department of Health Services; 2017.

[20] Social health security program operation rules (second amendment). Kathmandu: Social Health Security Deveopment Committee; 2017.

[21] Government of Nepal. Social health security programme: Standard operating procedures. Kathmandu: Social Health Security Development Committee; 2016.

[22] Foreign exchange rates. https://www.nrb.org.np/fxmexchangerate.php. Accessed September, 2018.

[23] Brief introduction of rural municipality and municipality. Kathmandu: Ministry of Federal Affairs and Local Development; 2017.

[24] National population and housing census 2011 (National report). Kathmandu, Nepal: National Planning Commission Secretariat, Central Bureau of Statistics; 2012.

[25] Bennet L, Dahal D, Govindasamy P. Caste, ethnic and regional identity in Nepal: further analysis of the 2006 Demographic and Health Surveys. Calverton, Maryland, USA: Macro International Inc;2008.

[26] Mainali D. Community based health insurance practices in Mangalbare primary health care centre, Morang district [Thesis]. Kathmandu: Tribhuwan University, Institute of Medicine; 2011.

[27] MOHP. Nepal demographic and health survey 2011. Kathmandu, Nepal: Ministry of Health and Population, New ERA, and ICF International; 2012.

[28] Andersen R, Newman JF (2005). Societal and individual determinants of medical care utilization in the United States. Milbank Q.

[29] Andersen RM (1995). Revisiting the behavioral model and access to medical care: does it matter?. J Health Soc Behav.

[30] Aregbeshola BS, Khan SM (2018). Predictors of enrolment in the National Health Insurance Scheme among women of reproductive age in Nigeria. Int J Health Policy Manag.

[31] Jehu-Appiah C, Aryeetey G, Spaan E, de Hoop T, Agyepong I, Baltussen R (2011). Equity aspects of the National Health Insurance Scheme in Ghana: who is enrolling, who is not and why?. Soc Sci Med.

[32] De Allegri M, Kouyaté B, Becher H (2006). Understanding enrolment in community health insurance in sub-Saharan Africa: a population-based case-control study in rural Burkina Faso. Bull World Health Organ.

[33] Manortey S, Alder S, Crookston B, Dickerson T, VanDerslice J, Benson S (2014). Social deterministic factors to participation in the National Health Insurance Scheme in the context of rural Ghanaian setting. J Public Health Afr.

[34] Kusi A, Enemark U, Hansen KS, Asante FA (2015). Refusal to enrol in Ghana’s National Health Insurance Scheme: is affordability the problem?. Int J Equity Health.

[35] Alatinga KA, Williams JJ (2015). Towards universal health coverage: exploring the determinants of household enrolment into National Health Insurance in the Kassena Nankana District, Ghana. Ghana Journal of Development Studies.

[36] Vyas S, Kumaranayake L (2006). Constructing socio-economic status indices: how to use principal components analysis. Health Policy Plan.

[37] Filmer D, Pritchett LH (2001). Estimating wealth effects without expenditure data—or tears: an application to educational enrollments in states of India. Demography.

[38] Bursac Z, Gauss CH, Williams DK, Hosmer DW (2008). Purposeful selection of variables in logistic regression. Source Code Biol Med.

[39] Poverty in Nepal 2010-11. Kathmandu: Central Bureau of Statistics; 2011.

[40] Adhikari N, Wagle RR, Adhikari DR, Thapa P, Adhikari M (2018). Factors affecting enrolment in the community based health insurance scheme of Chandranigahapur hospital of Rautahat district. J Nepal Health Res Counc.

[41] Badu E, Agyei-Baffour P, Ofori Acheampong I, Preprah Opoku M, Addai-Donkor K (2018). Households sociodemographic profile as predictors of health insurance uptake and service utilization: a cross-sectional study in a municipality of Ghana. Adv Public Health.

[42] Chankova S, Sulzbach S, Diop F (2008). Impact of mutual health organizations: evidence from West Africa. Health Policy Plan.

[43] Vellakkal S (2013). Determinants of enrolment in voluntary health insurance: evidences from a mixed method study, Kerala, India. International Journal of Financial Research.

[44] Saavedra M (2017). Children’s health insurance, family income, and welfare enrollment. Child Youth Serv Rev.

[45] Aggarwal A (2011). Achieving equity in health through community-based health insurance: India’s experience with a large CBHI programme. J Dev Stud.

[46] Mhere F (2013). Health insurance determinants in Zimbabwe: case of Gweru urban. J Appl Bus Econ.

[47] Parmar D, Souares A, De Allegri M, Savadogo G, Sauerborn R (2012). Adverse selection in a community-based health insurance scheme in rural Africa: implications for introducing targeted subsidies. BMC Health Serv Res.

[48] McIntyre D. Learning from experience: Health care financing in low-and middle-income countries. Geneva: Global forum for health research Geneva; 2007.

[49] Rajkotia Y, Frick K (2012). Does household enrolment reduce adverse selection in a voluntary health insurance system? Evidence from the Ghanaian National Health Insurance System. Health Policy Plan.

[50] Wang H, Zhang L, Yip W, Hsiao W (2006). Adverse selection in a voluntary Rural Mutual Health Care health insurance scheme in China. Soc Sci Med.

[51] Dong H, Kouyate B, Cairns J, Sauerborn R (2004). Differential willingness of household heads to pay community-based health insurance premia for themselves and other household members. Health Policy Plan.

[52] Kapologwe NA, Kagaruki GB, Kalolo A (2017). Barriers and facilitators to enrollment and re-enrollment into the community health funds/Tiba Kwa Kadi (CHF/TIKA) in Tanzania: a cross-sectional inquiry on the effects of socio-demographic factors and social marketing strategies. BMC Health Serv Res.

[53] Iqbal M, Chowdhury AH, Mahmood SS, Mia MN, Hanifi S, Bhuiya A (2017). Socioeconomic and programmatic determinants of renewal of membership in a voluntary micro health insurance scheme: evidence from Chakaria, Bangladesh. Glob Health Action.

[54] Boateng D, Awunyor-Vitor D (2013). Health insurance in Ghana: evaluation of policy holders’ perceptions and factors influencing policy renewal in the Volta region. Int J Equity Health.

